# Differential effects of dopaminergic medication and subthalamic deep brain stimulation on gait in Parkinson's disease: A two-case report

**DOI:** 10.1016/j.prdoa.2026.100511

**Published:** 2026-10-12

**Authors:** Lea Marie-Sophie Cordes, Sila Gül Bozkurt, Sihai Liu, Lukas Mödl, Peter Vajkoczy, Hendrik Schmidt, Philipp Spindler

**Affiliations:** a Julius Wolff Institute, Berlin Institute of Health at Charité – Universitätsmedizin Berlin | Berlin, Germany; b Department of Neurosurgery, Charité – Universitätsmedizin Berlin | Berlin, Germany; c Institute of Biometry and Clinical Epidemiology, Charité – Universitätsmedizin Berlin | Berlin, Germany

**Keywords:** Parkinson's disease, Gait, Deep brain stimulation, Dopaminergic medication, Case report

## Abstract

**Introduction:**

In Parkinson's disease (PD), interactions between medication (Med) and deep brain stimulation (DBS) across gait domains and in individuals with distinct clinical profiles remain incompletely understood. This study conducted detailed within-subject gait analyses in two individuals with PD across four conditions: (1) DBS ON/Med ON, (2) DBS OFF/Med ON, (3) DBS ON/Med OFF, and (4) DBS OFF/Med OFF, comparing results with healthy reference data.

**Case 1:**

Due to fluctuations in effectiveness that could no longer be treated with oral medication, accompanied by pronounced dyskinesia and insufficient tremor control, decision for subthalamic nucleus (STN) stimulator implantation was made. The patient continued to receive medication. STN-DBS primarily optimized axial control and intersegmental coordination, substantially increasing walking speed, while dopaminergic medication provided limited additional benefit.

**Case 2:**

The patient noticed increasing bradykinesia of the left leg, later accompanied by rigidity and resting tremor, and subsequently on the right side. He suffered from severe freezing episodes. Under dopamine agonists, he experienced nightmares and pseudohallucinations. Thus, STN stimulator implantation was performed. The patient continued to receive medication. STN-DBS improved posture and alignment, bringing kinematics closer to those of healthy controls, while the effects of combined STN-DBS and medication appeared more complementary than in Case 1.

**Conclusion:**

Results demonstrated differential and partially complementary effects of medication and stimulation across gait domains. Clinically, inter-individual variability should be considered. The DBS ON/Med ON condition did not consistently provide maximal improvement across all outcomes, highlighting the value of comprehensive gait assessment for individualized therapy optimization.

## Highlights

• Results demonstrated clinically meaningful inter-individual variability.
• Medication and stimulation showed differential, partly complementary effects.
• The combined ON condition did not consistently yield maximal gait improvement.
• Findings highlight value of detailed gait assessment for patient-specific responses.

## 1 Introduction

Gait impairment is a core feature of Parkinson's disease (PD), contributing to disability, reduced quality of life (QoL), and increased fall risk [1], [2]. It emerges early, progresses over time, and depends on dopaminergic and non-dopaminergic mechanisms [2], [3], [4]. Deep brain stimulation (DBS) of the subthalamic nucleus (STN) enhances motor symptoms, including gait and postural instability, with reported improvements in axial control, intersegmental coordination, and gait variability, complementing the effects of dopaminergic medication [5], [6], [7], [8], [9], [10]. However, detailed within-subject comparisons across medication and stimulation states, particularly incorporating kinematic and kinetic gait analyses, remain scarce, leaving their interaction across gait domains and in individuals with distinct clinical profiles incompletely understood.

This two-case report investigates the effects of dopaminergic medication and STN-DBS, alone and combined, on spatiotemporal, kinematic, and kinetic gait parameters. By comparing two PD patients with differing clinical characteristics across four treatment conditions, and relating their gait patterns to healthy reference data, the primary aim of this study was to provide a descriptive biomechanical profiling of domain- and patient-specific treatment effects on gait rather than to test hypotheses, while highlighting the interpretative scope and limitations of detailed case-based analyses.

## 2 Patient information, clinical findings & therapeutic intervention

### 2.1 Case 1

The male patient was 55 years old and had a BMI of 31.8 kg/m^2^. Due to fluctuations in effectiveness that could no longer be treated with oral medication, accompanied by pronounced dyskinesia and insufficient tremor control, the surgery for STN stimulator implantation took place in May 2020. Bilateral directional 8-contact DBS leads (Boston Scientific; exact electrode model unavailable) were implanted in the STN. Stimulation was monopolar, with the implantable pulse generator case serving as the anode. Contacts 2, 3, and 4 (left) and 10, 11, and 12 (right) served as active cathodes, with stimulation distributed at 34%, 33%, and 33%, respectively. Amplitude, frequency, and pulse width are reported in Fig. 1. The patient continued treatment with levodopa/benserazide, piribedil, and extended-release levodopa/benserazide, with a levodopa equivalent dose (LED) of 475 mg/d [11]. His QoL improved post-surgery (Parkinson's Disease Questionnaire-39 (PDQ-39) [12] Summary Index: 20.4 pre-surgery; 13.8 at 24 months post-surgery). At the time of study participation, his current disease burden was mild (Movement Disorder Society-Unified Parkinson's Disease Rating Scale (MDS-UPDRS) [13] Part III: Motor Examination, total score: 18; Hoehn and Yahr stage: 2 [14]) (Fig. 1). The MDS-UPDRS and Hoehn and Yahr assessments were performed with both STN-DBS and medication ON.

### 2.2 Case 2

The male patient was 64 years old and had a BMI of 19.7 kg/m^2^. Over the disease course, the patient developed increasing bradykinesia of the left leg, later with rigidity and resting tremor, eventually also affecting the right side. He experienced severe, immediate freezing episodes. Under dopamine agonists, he developed nightmares and pseudohallucinations. Therefore, STN stimulator implantation was performed in March 2021. Bilateral directional 8-contact DBS leads (Boston Scientific; exact electrode model unavailable) were implanted in the STN. Stimulation was monopolar, with the implantable pulse generator case serving as the anode. Contacts 2, 3, and 4 (left) and 10, 11, and 12 (right) served as active cathodes, with stimulation distributed at 33%, 33%, and 34%, respectively. Amplitude, frequency, and pulse width are reported in Fig. 1. The patient continued treatment with entacapone, levodopa/carbidopa, alfuzosin, and finasteride, with a LED of 665 mg/d. His QoL improved post-surgery (PDQ-39 Summary Index: 16 pre-surgery; 6.5 at 24 months post-surgery). At the time of study participation, his current disease burden was very low (MDS-UPDRS Part III: Motor Examination, total score: 1; Hoehn and Yahr stage: 0) (Fig. 1). As in Case 1, both assessments were performed with STN-DBS and medication ON.

### 2.3 Reference data

As a reference, we used data from 19 healthy male participants (age: 37.9 ± 12.2 years; BMI: 23.6 ± 2.3 kg/m^2^) [16]. They represented a well-defined asymptomatic subgroup of participants from the Berlin Back Study, with no history of back, pelvic, or hip pain or related surgery, no morphological abnormalities of the lumbar spine, and good overall health status as assessed using the SF-36 questionnaire [16], [17].

**Fig. 1:**
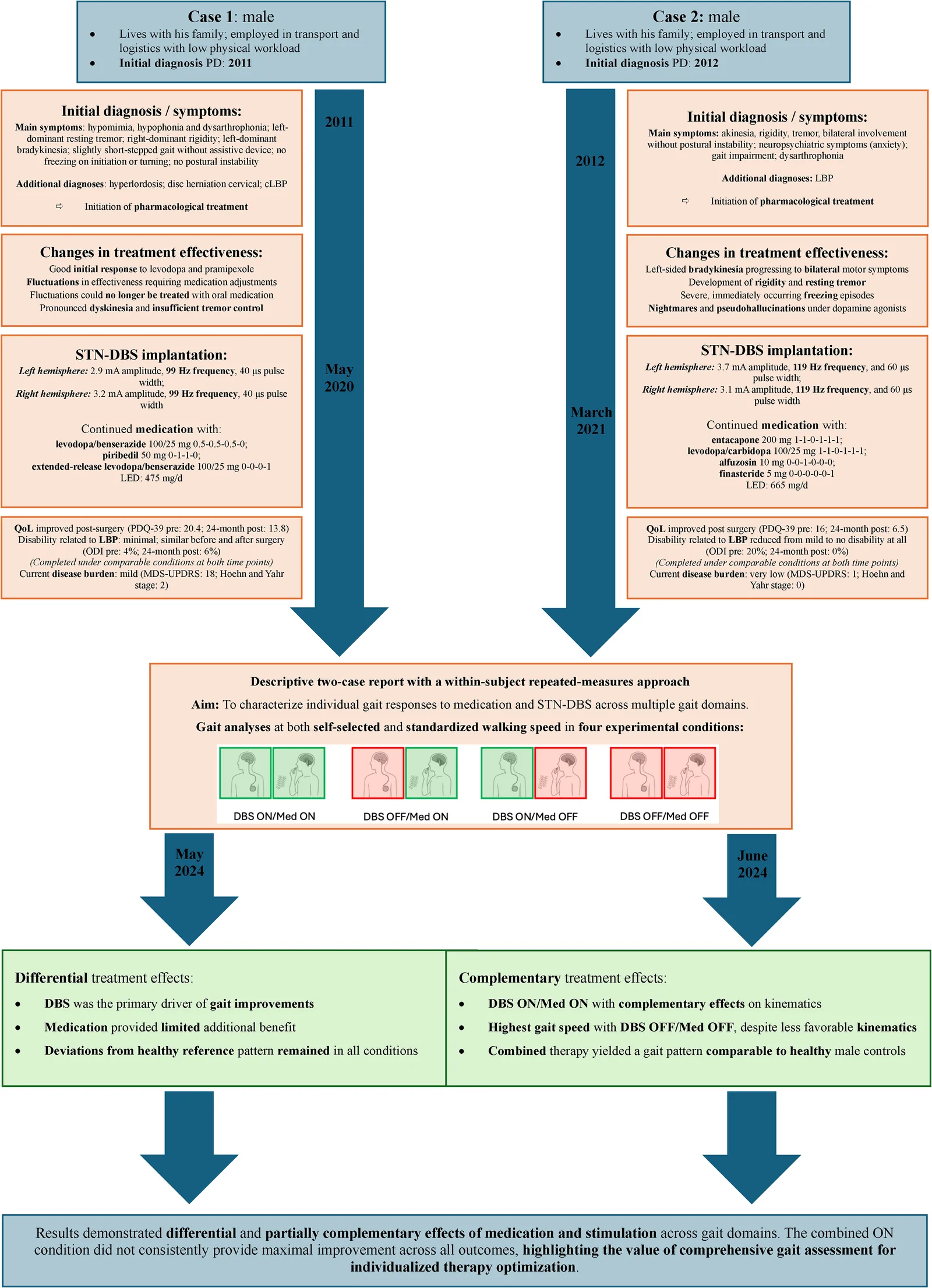
Timeline. Initial diagnosis, disease progression, treatments, diagnostic intervention, and its outcomes, separately for Cases 1 and 2. PD: Parkinson's Disease; STN-DBS: Subthalamic Nucleus Deep Brain Stimulation; cLBP: chronic Low Back Pain; LBP: Low Back Pain; LED: Levodopa Equivalent Dose; QoL: Quality of Life; PDQ-39: Parkinson's Disease Questionnaire-39; MDS-UPDRS: Movement Disorder Society-Unified Parkinson's Disease Rating Scale; ODI [15]: Oswestry Disability Index; DBS: Deep Brain Stimulation; Med: Medication.

## 3 Diagnostic assessment

### 3.1 Diagnostic testing & changes in therapeutic intervention

This study was designed as a descriptive two-case report with a within-subject repeated-measures approach. Two patients with PD who had previously undergone bilateral STN-DBS implantation were examined under four experimental conditions: (1) stimulation and medication on (DBS ON/Med ON), (2) stimulation off and medication on (DBS OFF/Med ON), (3) stimulation on and medication off (DBS ON/Med OFF), and (4) stimulation and medication off (DBS OFF/Med OFF). The order of conditions was predefined and documented to minimize bias and to allow transparent interpretation of potential order effects.

Atrsaei et al. [18] reported pronounced gait-related levodopa effects approximately 3 h after intake, although treatment responses vary considerably between individuals and may also depend on the medication type and formulation. Therefore, approximately 3 h after medication intake was considered a plausible Med ON time point, while also accounting for practical scheduling constraints. On the morning of testing, Case 1 received half a tablet of levodopa/benserazide (100/25 mg), while Case 2 received entacapone (200 mg) and levodopa/carbidopa (100/25 mg) (Fig. 1). Med OFF timing was based on the patients' self-reported duration of the morning ON state and was set at approximately 5 h after intake. At these later time points, the acute medication effect was assumed to have substantially declined. Accordingly, Med ON and OFF states were operationally defined, and residual pharmacological effects cannot be excluded.

The timing of the assessments was aligned with the patients' usual medication schedules and adjusted when necessary to fit the study protocol. In Case 1, the first medication intake was postponed to 7:30 a.m., and conditions 1–4 were assessed approximately 3 h, 3.5 h, 5 h, and 5.5 h after intake, respectively. In Case 2, the first medication intake occurred at 8:00 a.m., and conditions 1–4 were assessed approximately 3 h, 3.5 h, 6 h, and 6.5 h after intake, respectively. The longer wash-out period in Case 2 was chosen because the patient still perceived a Med ON state 5 h after intake, but no longer after 6 h.

DBS ON and OFF conditions were implemented by adjusting stimulation settings during the measurement session, allowing a clinically feasible wash-in and wash-out interval while balancing patient tolerability and symptom burden. The DBS wash-out interval between conditions 1 and 2, 3 and 4 was 15 minutes. Patients were unable to tolerate a longer wash-out period due to worsening of symptoms. DBS settings remained unchanged between the two DBS ON assessments.

Gait analyses were performed in a Gait Real-time Analysis Interactive Lab (GRAIL; Motek Medical B.V., Houten, the Netherlands), comprising an instrumented dual-belt treadmill (MotekForceLink), 14 motion-capture cameras (Vero v2.2; Vicon Motion Systems Ltd., Oxford, UK), and a virtual environment. Ground reaction forces (GRFs) were sampled at 1000 Hz and marker trajectories at 100 Hz. Motion tracking was conducted using 60 passive retroreflective markers (Prophysics AG, Kloten, Switzerland) [16]. Marker and force data were processed in Vicon Nexus 2.15.0 (Vicon Motion Systems Ltd., Oxford, UK) and subsequently analyzed in MATLAB R2024b (The MathWorks Inc., Natick, MA, USA). After gap filling, the Plug-in Gait Dynamic pipeline was applied. Vertical GRF signals were low-pass filtered at 6 Hz using a fourth-order Butterworth filter, and gait events were identified from a 10-N vertical GRF threshold [19]. Only steps with an unambiguous single-foot contact on one force plate were retained, while crossover steps were discarded. All valid gait cycles obtained from three 30-s trials were included in the calculation of the averaged waveforms, resulting in an average of 96 ± 10 gait cycles (range: 86–116) for Case 1 and 93 ± 27 gait cycles (range: 49–151) for Case 2 per condition and leg at both speeds.

Following a six-minute familiarization phase at self-selected walking speed, three 30-s gait recordings were obtained. If a substantial number of crossover steps occurred, additional trials were recorded. The treadmill speed was subsequently adjusted to a standardized speed of 4 km/h, if feasible, followed by a one-minute familiarization period and three additional 30-s recordings. This protocol was applied in each of the four experimental conditions. As not all conditions allowed safe and continuous walking at standardized speed, analyses focused primarily on self-selected speed, with standardized-speed trials reported descriptively to assess feasibility and tolerability. Self-selected treadmill speed was determined by asking participants to choose a speed that felt natural and reflected their usual everyday walking pace, with treadmill speed adjusted accordingly by the research staff. Leg dominance was determined by self-report based on the leg participants would preferentially use to kick a ball [20]. Both participants identified the right leg as dominant and the left leg as non-dominant.

Spatiotemporal gait parameters were summarized descriptively. Kinematic and kinetic data were visualized as averaged gait-cycle trajectories for each condition and interpreted relative to a healthy male reference dataset assessed using the same protocol [16]. A gait cycle was defined as from initial contact of one foot to initial contact of the same foot. Gait cycles were normalized from 0 to 100% and divided into the following phases: initial contact (IC) at 0%, loading response (LR) from 0 to 12%, mid stance (MSt) from 12 to 31%, terminal stance (TSt) from 31 to 50%, pre-swing (PSw) from 50 to 62%, initial swing (ISw) from 62 to 75%, mid swing (MSw) from 75 to 87%, and terminal swing (TSw) from 87 to 100% (Fig. 2, Fig. 3).

To account for speed dependence when interpreting gait outcomes, our interpretation was based on two complementary approaches. First, changes in spatiotemporal parameters were interpreted in relation to changes in gait speed. Second, gait performance was assessed holistically by integrating spatiotemporal, kinematic, and kinetic parameters. As range of motion (RoM) and peak values are themselves influenced by walking speed, we focused on the entire kinematic and kinetic waveforms rather than discrete outcome measures, allowing us to evaluate temporal and curve-shape-related deviations from reference data as well as changes between conditions. Because the analyses primarily focused on self-selected walking speed, patient and reference data were not speed matched. The mean gait speed of the reference group was 4.4 ± 0.9 km/h.

Given the descriptive nature of this two-case report, all gait variables were considered exploratory and were interpreted descriptively.

### 3.2 Diagnostic challenges

The protocol involved temporary withdrawal of therapeutic support, particularly STN-DBS deactivation, which may cause discomfort and increase assessment burden. The order of conditions was selected to account for the expected medication wash-out period while minimizing the time participants spent with reduced medication efficacy and at the same time ensuring an adequate approximation of the Med OFF state.

## 4 Outcomes

### 4.1 Clinician and patient-assessed outcomes

Case 1*:*

Under DBS ON conditions, the patient walked continuously and safely at self-selected speed. In DBS OFF conditions, the patient walked considerably slower and his tremor, which was particularly visible in his arms and hands (left-predominant), reappeared, requiring a reduced standardized walking speed of 2 km/h (Supplementary video S1).

At self-selected walking speed, DBS ON conditions were associated with higher gait speed, slightly lower total double support and smaller step width, a larger step length, and relatively lower cadence compared with DBS OFF conditions (Table 1). Kinematic analyses revealed a more upright trunk orientation and improved axial coordination under STN-DBS. Despite these improvements, residual deviations from healthy reference patterns remained, particularly at the hip and knee joints. The joint loadings were affected, however, hip and knee joint moments differed only modestly between conditions (Fig. 2 and Supplementary Material 1).

**Table 1:** Spatiotemporal parameters Case 1. Mean values of spatiotemporal parameters of Case 1 in each condition.

Case 1	Gait speed (self-selected) (in km/h)	Step width (in m)	Step length (in m)	Double step length (in m)	Cadence (step/min)	Total double support (%)	Stance (%)	Swing (%)
DBS ON / Med ON	3.20	0.17	0.55	1.07	96	34	67	33
DBS OFF / Med ON	2.52	0.21	0.43	0.90	89	37	68	32
DBS ON / Med OFF	3.31	0.19	0.53	1.06	101	34	66	34
DBS OFF / Med OFF	2.00	0.20	0.34	0.74	85	40	69	31

**Fig. 2:**
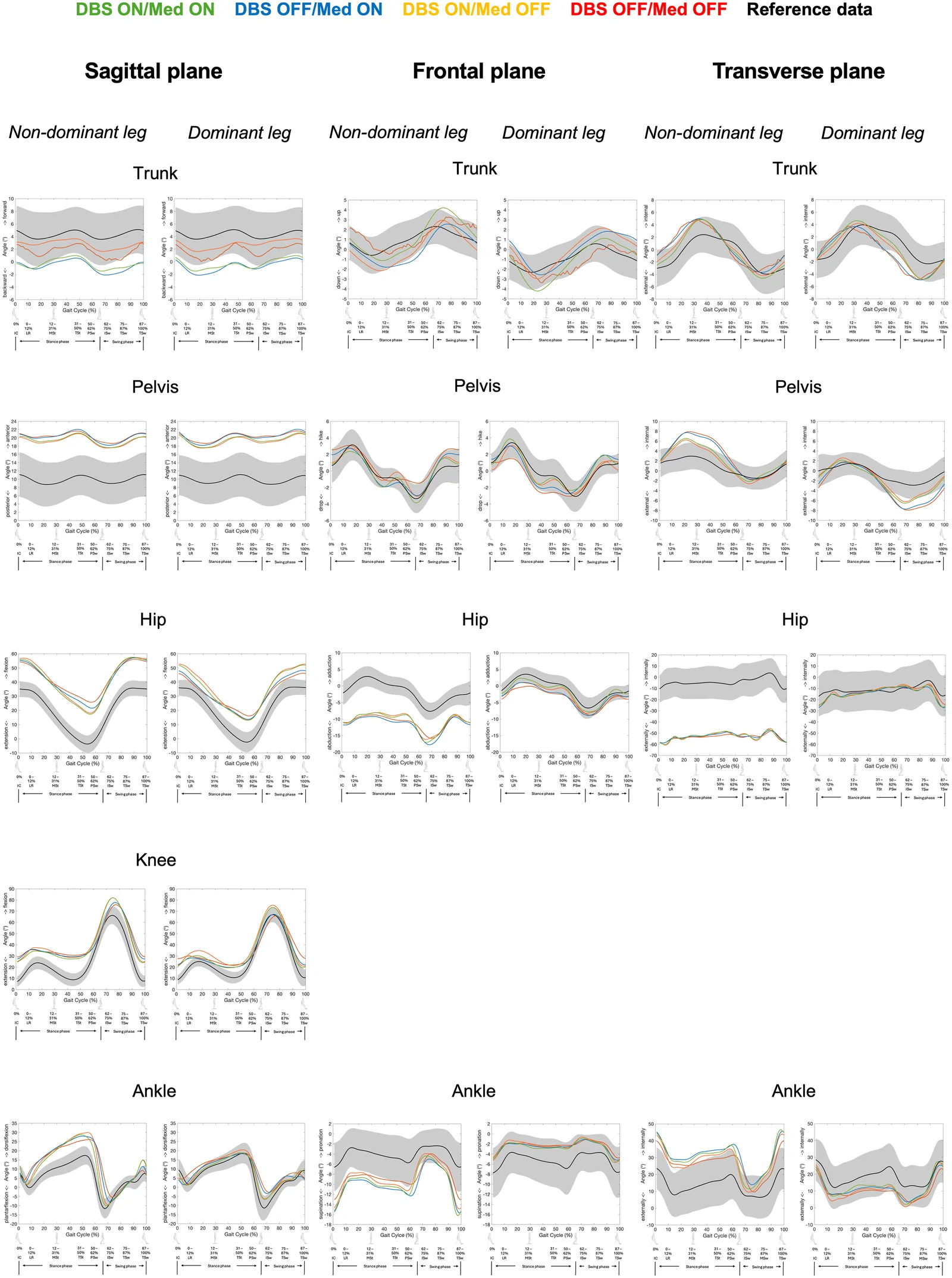
Kinematic gait parameter Case 1. Kinematic mean pattern (angles in °) for trunk, pelvis, and hip, knee and ankle joints in all four conditions: DBS ON/Med ON (solid green line); DBS OFF/Med ON (solid blue line); DBS ON/Med OFF (solid orange line); DBS OFF/Med OFF (solid red line); and reference data (solid black line: mean; shaded grey area: standard deviation). Below the waveform plots, a schematic representation of the gait cycle and its respective phases is shown. (For interpretation of the references to colour in this figure legend, the reader is referred to the web version of this article.)

Case 2*:*

Unlike Case 1, this patient walked at both self-selected and predefined standardized speed of 4 km/h across all conditions. No safety issues were observed, indicating preserved functional reserve despite disease duration.

Spatiotemporal gait parameters showed higher walking speeds in the DBS ON conditions and the DBS OFF/Med OFF condition, primarily reflecting increased cadence while step length remained largely unchanged (Table 2). In the DBS OFF/Med ON condition, walking speed and step length were lowest, whereas cadence was relatively higher than in all other conditions. The DBS ON conditions improved posture and body alignment, resulting in overall kinematics and kinetics more similar to those of healthy controls (Fig. 3 and Supplementary Material 1), with the combined DBS ON/Med ON condition appearing to show slightly greater complementary effects on kinematics than in Case 1.

**Table 2:** Spatiotemporal parameters Case 2. Mean values of spatiotemporal parameters of Case 2 in each condition.

Case 2	Gait speed (self-selected) (in km/h)	Step width (in m)	Step length (in m)	Double step length (in m)	Cadence (step/min)	Total double support (%)	Stance (%)	Swing (%)
DBS ON / Med ON	3.88	0.19	0.64	1.28	101	33	67	33
DBS OFF / Med ON	3.49	0.16	0.57	1.13	103	33	67	33
DBS ON / Med OFF	4.25	0.14	0.65	1.29	109	31	66	34
DBS OFF / Med OFF	4.54	0.13	0.65	1.30	117	31	66	34

**Fig. 3:**
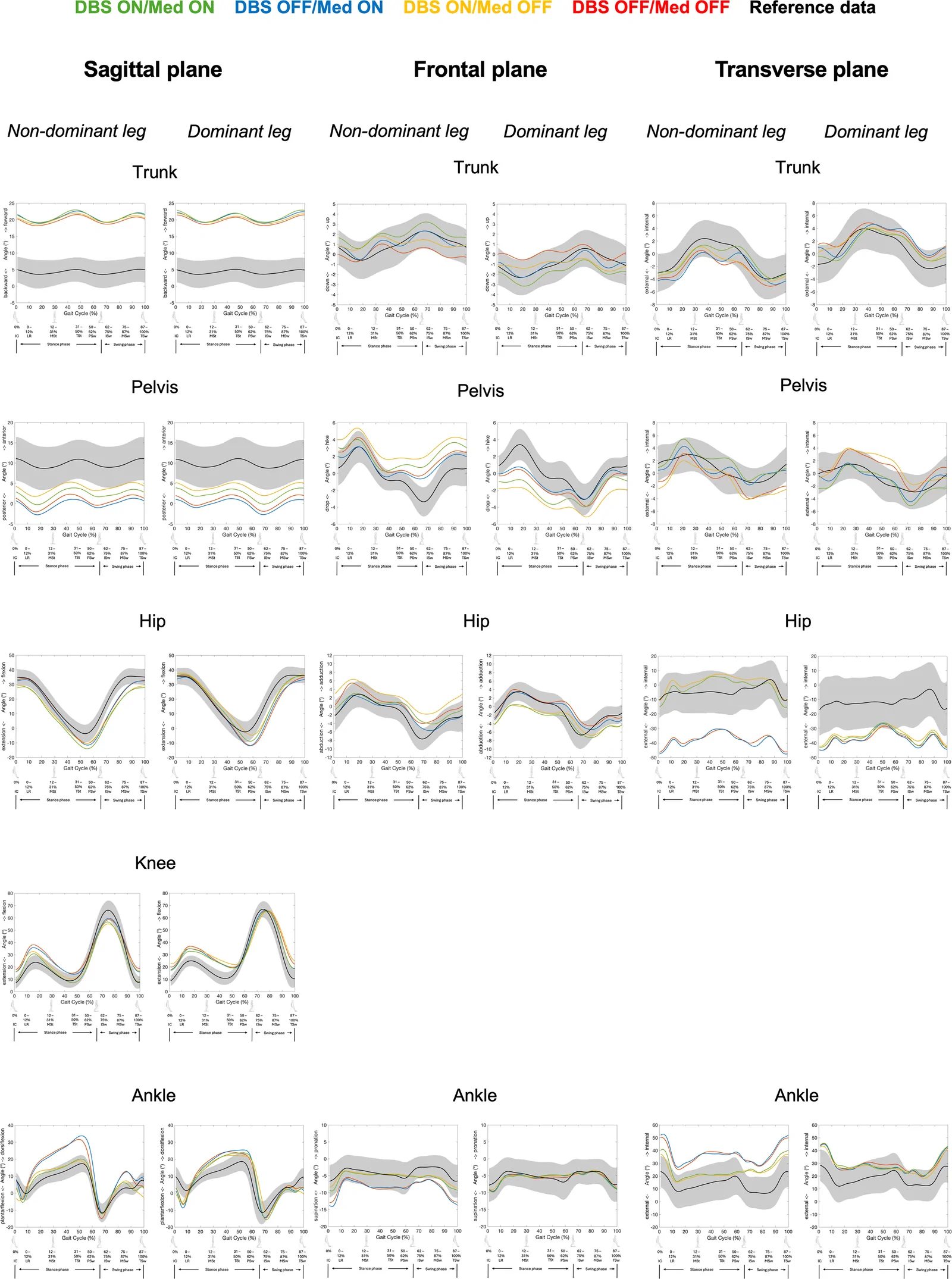
Kinematic gait parameter Case 2. Kinematic mean pattern (angles in °) for trunk, pelvis, and hip, knee and ankle joints in all four conditions: DBS ON/Med ON (solid green line); DBS OFF/Med ON (solid blue line); DBS ON/Med OFF (solid orange line); DBS OFF/Med OFF (solid red line); and reference data (solid black line: mean; shaded grey area: standard deviation). Below the waveform plots, a schematic representation of the gait cycle and its respective phases is shown. (For interpretation of the references to colour in this figure legend, the reader is referred to the web version of this article.)

### 4.2 Intervention adherence & tolerability

An investigator remained beside the treadmill to ensure safety, maintain communication, and control speed. Recordings were only initiated or continued if the patient provided positive feedback regarding the overall situation.

Case 1 reported that 4 km/h was not achievable in the DBS OFF conditions, thus standardized speed was reduced to 2 km/h.

## 5 Discussion

### 5.1 Strengths and limitations

The descriptive nature and small sample size preclude generalization. Because the order of conditions was dictated by medication wash-out requirements and patient tolerability, randomization was not feasible. Consequently, residual carry-over effects between conditions cannot be excluded and may have influenced gait performance. Accordingly, conclusions are limited to qualitative and mechanistic interpretation.

Although freezing of gait (FOG) represented an important clinical feature, it was not assessed using a standardized FOG-specific outcome measure. Therefore, conclusions regarding treatment effects on freezing remain limited.

However, from a clinical perspective, these cases highlight the potential value of detailed gait assessment for understanding patient-specific treatment responses. Such case-based analyses can help explain why patients experience different functional outcomes under similar therapeutic interventions.

### 5.2 Discussion of relevant literature

Case 1 and Case 2 demonstrated only slightly different responses to STN-DBS and medication, with modestly more complementary effects of combined STN-DBS and medication in Case 2. In Case 1, STN-DBS was the primary contributor to improvements in gait speed, axial posture, and intersegmental coordination, whereas medication provided little additional benefit and occasionally appeared to worsen gait performance. This pattern resembles the subgroup described by Barbosa et al. [21], in whom FOG improved with stimulation but worsened with levodopa. Furthermore, the addition of levodopa to otherwise effective stimulation was associated with an increase in FOG episodes. Although FOG was not specifically assessed in our patients, a comparable dissociation was observed at the level of overall gait performance and axial symptoms.

Despite treatment-related improvements, in Case 1 a stooped posture characterized by persistent hip and knee flexion remained present across all conditions. Additional deviations from healthy male reference patterns could potentially reflect patient-specific habitual, structural, or compensatory adaptations associated with the predominantly left-sided symptomatology rather than direct treatment effects. In contrast, the DBS ON conditions in Case 2 resulted in improved segmental control, posture, and joint loading patterns, yielding a gait pattern comparable to healthy male controls. Consistent with previous reports, medication-related improvements in trunk and pelvis control may have contributed to the somewhat more complementary effects of combined STN-DBS and medication observed in Case 2 [6], [22].

Beyond the modest differences in effects on gait performance observed between Cases 1 and 2, previous studies have reported substantial inter-individual variability in gait-related treatment effects in PD. In another subgroup reported by Barbosa et al. [21], FOG worsened with stimulation but improved with levodopa, and levodopa restored a more favorable motor response when combined with stimulation. Stimulation-induced gait impairments may result from the spread of stimulation to neural structures neighboring the STN [21]. Further potential explanations for the observed differences between PD patients include variations in disease phenotype, freezing propensity, symptom lateralization, baseline postural control, and individual stimulation response characteristics. In the present report, these factors were not systematically assessed and therefore remain speculative. Nevertheless, they may contribute to the substantial heterogeneity of gait-related treatment responses reported in the literature.

Interpretation of gait outcomes should consider the influence of walking speed, as changes in speed can affect spatiotemporal, kinematic, and kinetic parameters and thereby influence the interpretation of pathological gait patterns [23], [24]. Notably, in Case 2 the highest walking speed was observed in the DBS OFF/Med OFF condition, whereas kinematic and kinetic patterns were generally more similar to healthy reference data during DBS ON conditions. This observation illustrates that higher walking speed does not necessarily correspond to a more physiological gait pattern and underscores the importance of multidimensional gait assessment when evaluating treatment effects. The higher walking speed observed in the DBS OFF/Med OFF condition in Case 2 is consistent with findings suggesting that treadmill walking may act as an external pacemaker and stabilizes gait in some patients [25]. Together with evidence supporting treadmill training combined with cognitive tasks [2], [26], this finding suggests potential value in further investigating interventions targeting both motor and cognitive aspects of gait and their translation into sustained benefits in everyday life activities. Although treadmill and overground walking should not be considered fully equivalent, familiarization can reduce initial treadmill-specific adaptation effects. A familiarization period of approximately six minutes has been suggested to achieve stable performance across a range of gait parameters, although optimal familiarization times in neurological populations such as PD remain unclear [27]. In PD, treadmill walking may additionally act as an external pacemaker and thereby modify gait characteristics [25]. Nevertheless, findings from Mirelman et al. [26] suggest that treadmill-based training effects can extend beyond the treadmill, including improvements in overground gait speed and, particularly when combined with cognitively demanding training, obstacle negotiation and fall risk.

Furthermore, stimulation frequency may substantially influence gait outcomes because different frequencies modulate distinct neural networks [21], [22], [28]. Since the mechanisms underlying gait heterogeneity and the contribution of PD phenotypes remain incompletely understood [9], future studies could investigate responses to medication and STN-DBS in a phenotype-specific manner [29]. Moreover, it may be of interest to examine in greater detail the differential effects on muscle activity during walking [30]. Overall, the assumption of a universally synergistic effect of medication and STN-DBS should be interpreted cautiously, as treatment responses appear highly patient-specific, emphasizing the need for individualized therapeutic approaches and detailed gait assessment [31].

### 5.3 Primary take away

This two-case report demonstrates that STN-DBS was associated with greater improvements in axial control, intersegmental coordination, and gait organization, whereas medication appeared to provide only modest additional benefits. These observations should be interpreted as exploratory and hypothesis-generating. However, interindividual differences underscore heterogeneity of gait control mechanisms in PD and illustrate why group-level averages may obscure clinically meaningful individual patterns. The DBS ON/Med ON condition did not consistently provide maximal improvement, highlighting the value of comprehensive gait assessment for individualized therapy optimization.

The following are the supplementary data related to this article.

Supplementary video S1

Supplementary material 1 Kinetic gait parameter Cases 1 and 2.

Supplementary material 2 CARE checklist.

## Ethical compliance statement

Written informed consent was obtained from all participants. The protocol was approved by the Ethics Committee of Charité - Universitätsmedizin Berlin (approval numbers: EA1/058/21, EA4/141/23). Written consent for publication and distribution of identifiable images/videos was obtained and is on file.

## Declaration of generative AI and AI-assisted technologies in the manuscript preparation process

During the preparation of this work the authors used ChatGPT in order to improve readability and language. After using this tool, the authors reviewed and edited the content as needed and take full responsibility for the content of the published article.

## Case reports

This research follows the CARE guidelines and the CARE checklist.

## CRediT authorship contribution statement

**Lea Marie-Sophie Cordes:** Writing – review & editing, Writing – original draft, Visualization, Software, Methodology, Investigation, Formal analysis, Data curation. **Sila Gül Bozkurt:** Writing – review & editing, Writing – original draft, Visualization, Methodology, Investigation, Formal analysis. **Sihai Liu:** Writing – review & editing, Investigation. **Lukas Mödl:** Writing – review & editing, Formal analysis. **Peter Vajkoczy:** Writing – review & editing, Supervision, Resources, Conceptualization. **Hendrik Schmidt:** Writing – review & editing, Supervision, Resources, Project administration, Methodology, Funding acquisition, Data curation, Conceptualization. **Philipp Spindler:** Writing – review & editing, Supervision, Resources, Project administration, Methodology, Conceptualization.

## Ethics declaration

Written informed consent to take part in the study and to publish the article has been obtained from all participants or their legal representatives. The privacy rights of participants have been observed.

This study was performed in compliance with relevant laws, regulatory frameworks and guidelines where the research took place.

This study was approved by the Ethics Committee of Charité - Universitätsmedizin Berlin.

(Approval No. EA1/058/21; EA4/141/23)

## Funding

This work is part of the Research Unit FOR5177 and was supported by the 10.13039/100005930German Research Foundation (SCHM 2572/11–2, SCHM 2572/13–2).

## Declaration of competing interest

The authors declare the following financial interests/personal relationships which may be considered as potential competing interests: (Hendrik Schmidt reports financial support was provided by German Research Foundation. If there are other authors, they declare that they have no known competing financial interests or personal relationships that could have appeared to influence the work reported in this paper).
