# Supplementary material for: Differential effects of dopaminergic medication and subthalamic deep brain stimulation on gait in Parkinson's disease: A two-case report
Source: Clin Park Relat Disord. 2026 Oct 12;15:100511. doi: 10.1016/j.prdoa.2026.100511 (PMC13764159; doi:10.1016/j.prdoa.2026.100511)
Supplement: Supplementary file 1 [file mmc2.pdf]

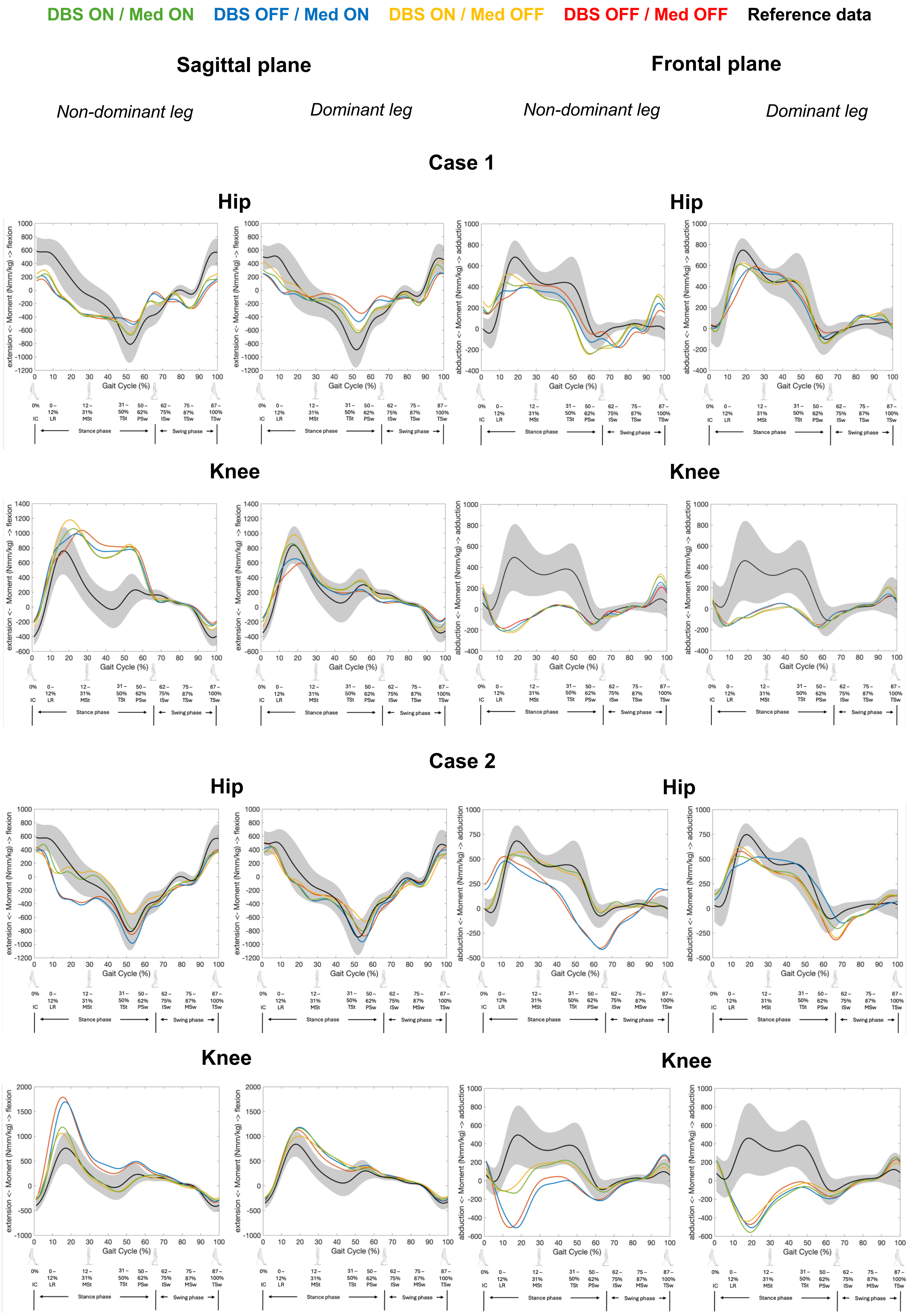
DBS ON / Med ON
DBS OFF / Med ON
DBS ON / Med OFF
DBS OFF / Med OFF
Reference data
Sagittal plane
Frontal plane
Non-dominant leg
Dominant leg
Non-dominant leg
Dominant leg
Case 1
Hip
Hip
Knee
Knee
Case 2
Hip
Hip
Knee
Knee
extension <- Moment (Nmm/kg) -> flexion
abduction <- Moment (Nmm/kg) -> adduction
Gait Cycle (%)
0% IC
0 – 12% LR
12 – 31% MSt
31 – 50% TSt
50 – 62% PSw
62 – 75% ISw
75 – 87% TSw
75 – 87% MSw
87 – 100% TSw
Stance phase
Swing phase

Supplementary material 1. Kinetic gait parameter of Case 1 and 2. Kinetic mean pattern (moments in Nmm/kg) for hip and knee joints in all four conditions: DBS ON/Med ON (solid green line); DBS OFF/Med ON (solid blue line); DBS ON/Med OFF (solid orange line); DBS OFF/Med OFF (solid red line); and reference data (solid black line: mean; shaded grey area: standard deviation). Below the waveform plots, a schematic representation of the gait cycle, divided into its respective phases is shown: IC: Initial contact, LR: Loading Response, MSt: Mid Stance, TSt: Terminal Stance, PSw: Pre-Swing, ISw: Initial Swing, MSw: Mid Swing, TSw: Terminal Swing.
